# Adaptation in Arctic circumpolar communities: food and water security in a changing climate

**DOI:** 10.3402/ijch.v75.33820

**Published:** 2016-12-13

**Authors:** James Berner, Michael Brubaker, Boris Revitch, Eva Kreummel, Moses Tcheripanoff, Jake Bell

**Affiliations:** 1 Alaska Native Tribal Health Consortium, Anchorage, AK, USA; 2 Institute of Economic Forecasts, Russian Academy of Sciences, Moscow, Russia; 3 Inuit Circumpolar Council, Ottawa, ON, Canada

**Keywords:** adaptation, food security, water security, vulnerable populations, local environmental observer, Rural Alaska Monitoring Program, traditional ecological knowledge

## Abstract

The AMAP Human Health Assessment Group has developed different adaptation strategies through a long-term collaboration with all Arctic countries. Different adaptation strategies are discussed, with examples mainly from native population groups in Alaska.

This paper focuses on adaptation to the environmental impact of climate change, with an emphasis on those small rural communities with a high dependence on local wildlife resources, stable permafrost, ice for winter subsistence hunting and other temperature-critical environmental or ecosystem characteristics. Regardless of the Arctic nation in which these small communities are located, they share many of the same vulnerabilities. These include a small resident population; little or no wage-based economy; a remote location, often with dependence on air or water transport at great expense; unreliable and expensive power generation; susceptibility to forest fire, flooding and coastal erosion of village sites by storms or flooding; and failure of key infrastructure owing to thawing permafrost, including loss of permafrost containment of tundra pond water sources and village sewage lagoons (1, 2).,

**Fig. 1:**
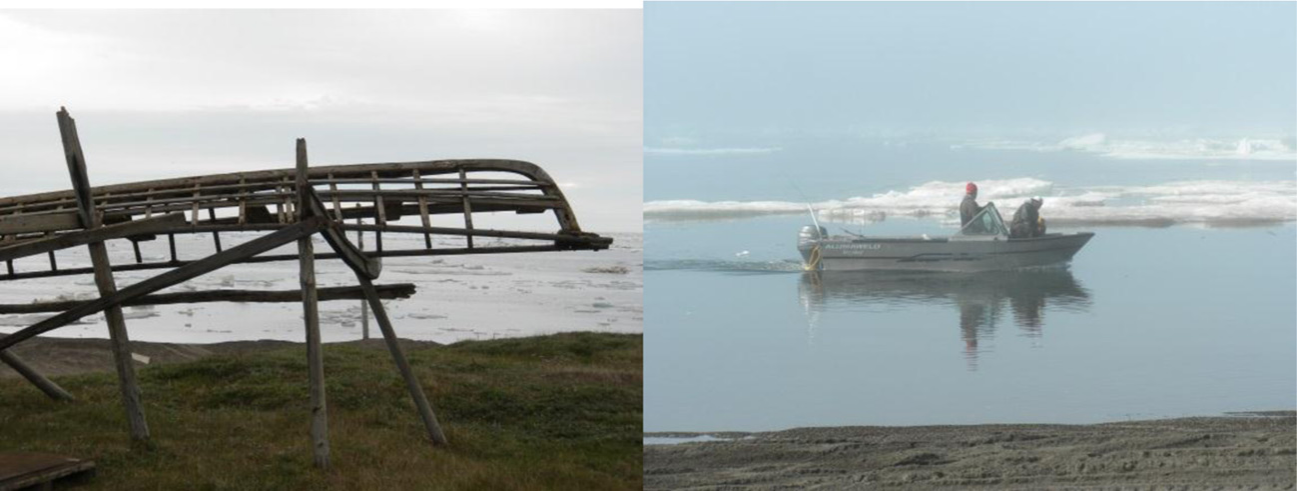
With climate change, a new whaling tradition is developing in the North Slope.

**Fig. 2:**
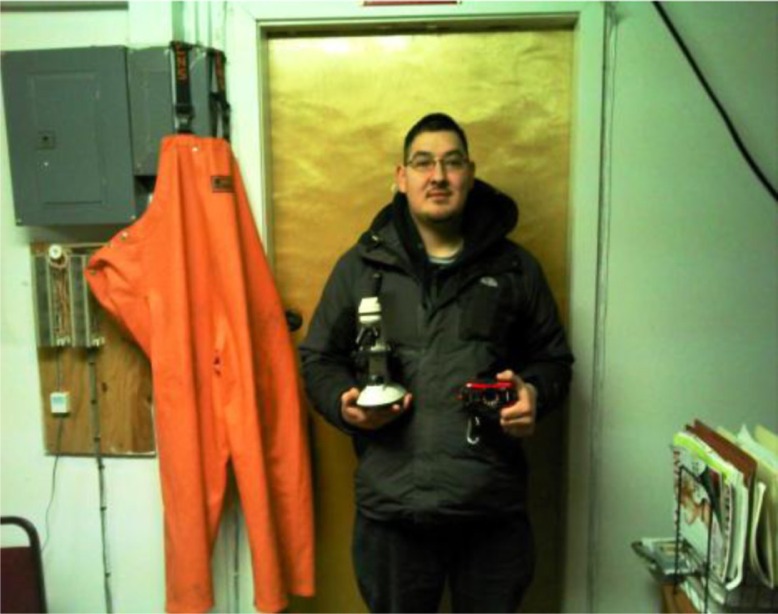
Aaron Merculief and local observer tools of trade.

For the purposes of this paper, *Adaptation* is defined as the ability of a small rural community to tolerate change imposed by climate regime change. The changes discussed are major environmental changes, which directly threaten the security of subsistence food and community water supply, and so threaten community sustainability. In this context, adaptation is the ability of the community to change, or adopt new behaviours, in order to avoid or minimise risk, and to be able to continue the most important cultural, economic and health-related benefits of the present way of living. The most recent assessment of the Intergovernmental Panel on Climate Change (IPCC) concluded with a major recommendation that all levels of government, in all regions, emphasise the development of adaptation strategies (3).

This discussion addresses climate-mediated impacts on remote rural communities. Some of these impacts are already occurring in parts of the Arctic, while other regions have experienced relatively few impacts. Adaptation activity is underway in some regions, but is still at the planning stage in others. Examples of community-based adaptation strategies are used to illustrate the wide range of strategies currently in use. Because this paper focuses on the impacts of environmental change, socio-economic impacts and changes in national policy are not addressed here. The often profound impacts on infrastructure, especially those related to permafrost and ice, are also not discussed. Readers interested in these topics are referred to the relevant sections of the Arctic Climate Impact Assessment (4, 5).

## Categories of environmental health threat

The major categories of environmental health threats emerging in the Arctic are climate change (principally climate warming), anthropogenic contaminants and zoonotic diseases (diseases of wildlife communicable to humans). These impacts, and the ways in which they interact, present an existential threat to small communities through their potential effects on subsistence food and community drinking water security. How communities are responding to such issues is important. Ideally, the response would include assessing the impacts of climate warming on infrastructure, food security and water security; prioritizing existing threats; identifying the most vulnerable residents; developing community-specific adaptation strategies designed to reduce risk to the most vulnerable subset of the population; and establishing metrics to monitor trends in established environmental threats and to detect emerging threats. Human biomonitoring is not addressed here as it is fully discussed in chapter 3 of the 2015 Arctic Human Health Assessment.

## Climate change in the Arctic region

Climate regime change in the circumpolar north varies widely as it is greatly affected by proximity to the Arctic Ocean, the Bering Sea, the North Pacific Ocean, and the northern extensions of the Atlantic Ocean. In addition, seasonal variation in the strength of the stable low atmospheric pressure fields (the Aleutian Low in the North Pacific and the Icelandic Low in the North Atlantic) and high atmospheric pressure fields (over Siberia and Arctic Canada) gives rise to very different regional weather patterns. The northern extension of the North Atlantic thermohaline circulation (known as the Gulf Stream) warms the Labrador Sea, and an eastern extension (known as the North Atlantic Drift) warms western Europe. Changes in water temperature around the eastern shore of the North Pacific (known as the Pacific Decadal Oscillation) result in significant differences in air temperature over adjacent land masses. There are many other cyclical atmospheric and oceanic changes that also drive environmental and ecological change, but these are not described here further. The annual Arctic Report Card (6) provides a recent discussion of Arctic climate trends, while the Arctic Climate Impact Assessment (7) gives an in-depth discussion of many of the underlying mechanisms.

## Anthropogenic contaminants

Environmental contaminants of anthropogenic origin are mostly produced and released by sources at lower latitudes and then transported to the Arctic by winds, ocean currents and rivers. After entering the northern environment, toxic heavy metals (such as mercury and lead) and persistent organic pollutants (POPs) enter the Arctic food chain and bioaccumulate through successively higher trophic levels. Tissue concentrations of lipid-soluble POPs, which have a long half-life in fat tissue, may be many hundreds of times higher in top predators than in organisms at the lowest trophic level, putting consumers of these top predators at risk from toxic effects.

Among the many toxic effects, a major concern is suppression of the immune system, making humans and animals more likely to develop an active infection.

## Zoonotic diseases

A number of microbial infections occur in both humans and the animals, which they harvest and consume; in healthy humans and animals, these infections are most often suppressed by the immune system and offer no health risk to the animal or human. However, under some circumstances, animals and humans can develop an active infection from a pathogen that has been suppressed for many years. This is usually associated with the occurrence of factors that affect the immune system such as a decrease in natural immunity with age; an immature immune system in infancy; changes in immunity during pregnancy, or during treatment with a medication that suppresses the immune system such as cancer chemotherapy or steroid medications; and exposure to high tissue levels of immunosuppressive contaminants.

The most common zoonotic infections are caused by three types of bacteria – *Brucella*, *Toxoplasma* and *Coxiella* – which are found in marine mammals, terrestrial mammals, birds and many other animals throughout the circumpolar north. There are many other zoonotic pathogens, which are common in some regions, but more localised in their distribution. New zoonotic pathogens are spreading the North as climate warming has made conditions more favourable. Examples include West Nile Virus in North America (which has reached the prairie provinces of Canada) and tick-borne encephalitis in northern Europe. Studies suggest that existing infectious threats may be present in greater numbers of subsistence animals and that pathogens new to the Arctic may continue to spread northward (8).

## Combined effects of climate warming, anthropogenic contaminants and zoonotic disease

A change in temperature may directly affect community infrastructure (including water treatment and sewage treatment facilities, power supply, and runways, harbours, roads and schools) and community food and drinking water security (defined here as adequate supply, adequate access and adequate information concerning food-borne threats such as contaminants and food-borne disease agents). However, the *combined effects* of climate warming, anthropogenic contaminants and zoonotic diseases also represent a significant risk to the security of subsistence food and drinking water supplies. Many circumpolar communities, particularly small rural communities, will need to develop monitoring and adaptation strategies to deal with these interacting risks.

- The warming climate has resulted in changes in ocean and atmospheric contaminant transport, which may have increased the movement of organohalogens and mercury from lower latitudes to the Arctic (9). Warmer water in freshwater lakes, tundra ponds and streams may result in greater bacterial methylation of mercury, and mercury released from thawing permafrost (10).
- Climate-mediated changes in the transport of anthropogenic contaminants to the circumpolar region may result in higher exposure in subsistence wildlife, which could increase the risk of immunosuppression and thus active zoonotic infection in these animals. This could result in risk to human consumers of infection and toxic effects from contaminant exposure (11).
- The warming climate is enabling southern plant, insect and animal species to expand their ranges further north, in some cases, into Arctic regions. These species may bring new zoonotic diseases with them. Higher winter temperatures in the Arctic may increase the winter survival of infected animals, raising the risk of hunter/consumer exposure.
- The northward expansion of new species has brought new water-borne diseases, such as tularaemia (8, 12), and warmer waters in tundra ponds and estuaries and nearshore ocean waters can support toxin-producing cyanobacteria and toxin-producing algal blooms (13).
- Longer Arctic summers and warmer winters, with less sea-ice cover, may increase the use of the Northern Sea Route by commercial shipping from northern Europe and western Russian ports (14), raising the possibility of new rat-borne infections from Europe to the North American Arctic. Tick-borne encephalitis is one such example (15).
- The warming climate may drive changes in the forage resources of subsistence species, and thus in the range, health and abundance of those subsistence species. The ecosystem changes could also impact on existing and newly emerging zoonotic pathogens of subsistence species.

## Developing a community-based adaptation strategy

The environmental impacts of climate warming, anthropogenic contaminants and zoonotic disease on subsistence food species and rural drinking water represent direct threats to the sustainability of small rural communities and create the need for community-based adaptation planning. There are four steps in the development of a community-specific adaptation strategy.

1. An assessment of the known, and potential, environmental threats. This may require the assistance of regional, tribal and/or government agencies, as well as academic institutions with this assessment capacity.
2. A community review of the data available and a community decision, taking into account traditional ecological knowledge (TEK), on prioritizing existing and potential threats and selecting the highest priority threats for further assessment. Communities rarely have in-depth information on the prevalence of zoonotic infections or tissue levels of contaminants in subsistence species and may require the assistance of regional wildlife managers to acquire the necessary data, such as water quality data from regional authorities for use as a historical baseline, long-term weather observations, permafrost data and other regional baseline data.
3. Once the relevant data have been obtained, adaptation planning can start. A critical part of adaptation planning involves identifying the subset of residents most at risk for the prioritized threats, which may require assistance from regional medical providers. The adaptation strategy should be directed at reducing risk for this group.
4. Plans for monitoring key indicators in order to follow the trends in threat development, and to identify newly emerging threats are the final steps in developing a community-specific adaptation strategy. Community-based monitoring will give the community trend data on increasing or decreasing risk from the prioritized threats, and will allow the adaptation plan to be modified accordingly. This activity may benefit from assistance by regional agencies with relevant monitoring capacity and the ability to train residents and provide equipment and supplies. The ANTHC has developed a community-based environmental monitoring program, the Rural Alaska Monitoring Program (RAMP), supported by a research grant from the US Environmental Protection Agency. The RAMP trains participating village hunters in the use of filter paper blood monitoring of harvested subsistence animals, and tests shellfish from village beaches for harmful algal toxins, as well as new vector-borne diseases moving north as species extend heir range. Many other metrics can also be monitored, as the village prioritizes the threats.
5. Data metrics in current use include long-term local weather data; environmental parameters, such as water temperature and water levels of nitrogen, mercury and phosphorus; permafrost temperature; river- and sea-ice data; filter paper sampling of animal blood by village hunters for the presence of antibodies to zoonotic infections (16), mercury (17) and other contaminants (18); biological toxin levels (such as from harmful algal blooms) in subsistence marine species; shoreline changes; and insect vector sampling for the presence of pathogenic bacteria.

A useful component of any adaptation strategy, or monitoring plan, is to use residents as local environmental observers (LEOs) to link new environmental events or observations with networks of regional wildlife management agencies, health care providers and academic consultants. This type of observer network has proved very useful for early detection of significant environmental events and conditions. In Alaska, the network of LEOs often provides the first warning of a significant environmental event, such as a mass wildlife mortality event in an otherwise unobserved remote area. The LEO network of observers has also noted the appearance of new species, and signs of new wildlife disease events, and can quickly communicate each observation via a network of Internet-connected LEOs (19).

## Managing the response to environmental threats

One of the most important benefits of the multistep process used for developing community-based adaptation strategies is that it enables residents to help plan, contribute TEK and participate in the investigation, and so build understanding of the most important environmental threats to their community. As part of the process, the entire community is involved in managing the response to the environmental threats. An equally important benefit is the recognition of vulnerable subsets of the population, and the development of strategies to reduce exposure, and thus risk, where exposure cannot be prevented. In terms of harvest plans, for example, this might involve harvesting younger and smaller seals to reduce the exposure of infants, children and pregnant women to mercury and organohalogens. It might involve changing food preparation practices for these populations, and for residents on chemotherapy, or those with chronic diseases, when a particular species is known to have a high prevalence of zoonotic exposure. Using informed adaptation plans, the health, cultural and economic benefits of the traditional subsistence diet can be preserved, and the risk to the most vulnerable residents can be understood and reduced. The following three sections provide examples of successful adaptation strategies, and an example of the critical role of an LEO network.

## Seasonal changes to whale hunting in Wainwright, Alaska

Coastal communities in the Arctic have long-established expertise in travelling on the frozen sea and using sea ice as a platform to harvest a wide range of food resources. In the Iñupiat village of Wainwright in Alaska, this includes hunting for bowhead whale, walrus, seal, polar bear, fish and birds. In Wainwright, a sustained west wind in winter will “ground” the sea ice and cause it to build into large stacked sheets attached to the sea floor. An east wind during spring will then create a break between the shore-fast ice and the floating sea ice, known as a “lead.” The lead provides a passage of open water through which whales pass on their northward migration. The grounded ice provides a platform to camp, launch boats and haul out the enormous whales for processing. In recent years, however, conditions have been changing. Warming has resulted in a decline in sea ice, particularly in the thick multi-year ice, and there has been a breakdown of the historical wind regimes that are crucial for safe travel and hunting conditions on the sea ice. Winds from the northwest and south have prevented the grounding of the ice and the formation of an accessible lead. The result is new challenges for ice-based hunting in Wainwright and other parts of the Arctic.

Starting in the 1980s, hunters in Wainwright began to notice changes in the sea ice and to discuss the need for entirely new hunting methods. Traditionally, Wainwright hunters pursued bowhead during spring, working off the ice shelf on the edge of the open water; launching skin boats when a whale was sighted and paddling in pursuit to harpoon a passing whale. Only a limited number of harpoon strikes are currently allowed by the International Whaling Commission, so each attempt must be taken with great care. In recent years, unusual wind and poor ice conditions have combined to prevent hunting, sometimes for weeks. In some years, ice conditions only improved after the whales had passed Wainwright. Therefore, in autumn 2011 with unused harpoon strikes available and open water across the Chukchi Sea, Wainwright hunters harvested their first autumn bowhead whale in the memory of current residents. It may even have been the first autumn bowhead harvested from Wainwright since the early American whaling era in the Arctic (late 1800s). To accomplish this, the hunters had had to acquire larger boats that could go further and handle the growing ocean swell generated by miles of wind across ice-free seas. If warming continues, boat-based hunting may also become common for spring hunting. The end result is a new and successful adaptation strategy, adding autumn sea-based hunting to the traditional spring ice-based hunting and increasing food security for one Arctic community Fig. 1.

## Maintaining food security – an example from the Canadian Arctic

Caribou herds are currently declining in several regions of Arctic Canada. For example, the George River migratory caribou herd, whose range spans Quebec and Labrador was probably around 800,000 animals 25 years ago, was counted to around 74,000 animals and declining in 2010, and later estimated to be only 20,000 animals. The low numbers caused the Government of Newfoundland and Labrador to impose a 5-year ban on harvesting in early 2013, and the Government of Quebec halted sports hunting indefinitely. Prior to the 5-year ban, the Nunatsiavut Government called on all beneficiaries of the Labrador Inuit Land Claims Agreement to suspend harvesting immediately for a 2-year period and encouraged other Aboriginal groups to do the same.

While the size of caribou herds appears to fluctuate as part of their population dynamics, climate change may represent an additional stress (www.caribou-ungava.ulaval.ca) that could weaken herds to the point of no recovery. A recent survey on Baffin Island caribou found that herds there are also severely reduced.

Inuit communities that rely on caribou as a staple country food are adapting in different ways. For example, Aboriginal groups in Quebec and Labrador formed the Ungava Caribou Aboriginal Roundtable to work on a conservation plan, while several Inuit populations are likely to be consuming more seal as a partial replacement for caribou in the diet. However, because contaminant levels in seals are generally higher than in caribou, this could result in increased contaminant exposure for some populations, unless they switch to less contaminated foods such as musk ox and anadromous Arctic char. Various studies are underway on whether climate change is causing higher levels of contaminants in marine mammals such beluga and seals. Concerns around contaminants and human health in the Canadian North prompted the creation of the Northern Contaminant Program in 1991, which has regularly measured and reported levels, sources and transport of contaminants in environment, biota and subsistence species in this region for many years (20).

## Applying local observation in response to food safety concerns

Climate change in Alaska is causing a wide range of impacts on the environment and on the health of animals and people (21). But in this vast and sparsely populated state, systems for monitoring changes in weather, landscape, biota and public health are limited. However, improvements in communications and local environmental capacity have led the Alaska Native Tribal Health Consortium (ANTHC) to develop a system for sharing information on environmental impacts and community health effects. This LEO network documents time- and location-specific events and encourages communication between communities, academic institutions and resource agencies to increase the understanding about climate and other drivers of change and to develop adaptation strategies. LEO applies traditional knowledge, western science and modern technology to achieve a robust and effective environmental health surveillance system.

LEO is an association of environmental and wildlife managers and health professionals located in tribal organisations in Alaska and western Canada. There are LEO network members in about 140 communities. They share observations about unusual and unique environmental events, which are then posted on public Google maps. Since the programme was initiated in January 2012, the network has compiled a database of observations on topics including extreme weather, floods, erosion, ice changes, permafrost thaw, invasive species, infrastructure damage, environmental contamination and changes in the health, range and behaviour of fish, insects, birds and wildlife. The utility of public observer networks for connecting remote communities with technical experts to help address food safety and other public health concerns is described in the section that follows.

## A village LEO investigates an alarming color change in the village harbor water

With the likelihood that climate change will continue to grow as a global public health challenge, it is important that communities have the capacity to monitor, respond and adapt to new events, impacts and health effects. The LEO network provides one possible template for engaging communities to perform environmental monitoring, improve communication and connect with technical experts and resources as required. It is a powerful tool for documenting local events and developing effective adaptation strategies for communities in the changing Arctic (22) Fig. 2.

Village-based environmental monitoring data can also be used for broader applications. For example, data on contaminant levels in animal tissue can lead to a better understanding of the impact of climate change on the transport and fate of contaminants within the Arctic, and on regional trends in contaminant levels. Such information can be used to indicate the efficacy of national and international programmes to reduce the production and release of contaminants. Another application of village-based environmental monitoring data concerns zoonotic diseases. Climate change is affecting the distribution of known pathogens within the Arctic and causing the emergence of pathogens new to the Arctic, and this is creating a need to make residents aware of the possible importance of modifying food preparation and storage methods. Regional public health and wildlife managers can then follow trends in pathogen prevalence in subsistence species. Archived biosamples should be preserved for baseline reference in identifying emergence of new pathogens or contaminants.

In September 2012, the Environmental Program Manager for the tribal government of St. George Island (and also the LEO for St. George) was informed that the water in the harbour had turned red. On St. George Island, residents harvest food locally from the land and sea. Their intimate knowledge of the climate, environment and biota translates into exceptional observational skills and a wealth of TEK. Consulting with local experts and elders, the LEO confirmed that there was no memory of this type of event ever having occurred in the past. Suspecting a harmful algal bloom and concerned about the safety of wild foods harvested from local waters for subsistence, he began to acquire information about the event. He collected surface and underwater photos (by placing his camera in a plastic bag and tying it to an oar), took a video of the harbour water and collected water samples. He then used the only microscope on the island, his daughter's “My First Microscope,” to examine the samples and find that the cause of the red harbour water was a living organism. He then took photomicrographs using a point and shoot digital camera and posted the observations, images and video on the LEO network website, including details of the event and comments about why the event was of local significance.

Once reviewed at the ANTHC Center for Climate and Health, the observation was transferred to a public, web-based Google map. The map link and a request for assistance was emailed to consultants at the University of Alaska Fairbanks (UAF), Institute of Marine Science and then by further referral, to consultants at the Woods Hole Oceanographic Institute (WHOI). Water samples collected by the LEO were sent to WHOI for genetic analysis and academic consultants used the data to provide a preliminary identification of the bloom source; not a dinoflagellate plankton such as is commonly associated with toxic red tides, but instead a harmless non-toxic ciliate called *Mesodinium rubrum*. Within a week of posting his observation, the LEO and the village had the answer, and the findings were distributed to St. George Island residents. The outcome and guidance for future events were posted on the original Google map and shared with the LEO membership via webinar. Based on concerns about harmful algal blooms in Alaska, a topic-based web map was then developed by the UAF, the WHOI and LEO to track related future events posted by the network.

## Conclusions and recommendations for addressing gaps in knowledge

The challenges posed by the combined impact of climate change, anthropogenic contaminants and zoonotic diseases on small, isolated Arctic communities will need to be met with adaptation strategies that aim to ensure effective responses to these challenges in order to maintain and enhance the stability of the community. Several examples of community-based responses have been presented in this paper. Some conclusions based on these examples include the following:

- The community impacts of Arctic warming differ across the circumpolar region and tend to be more severe in regions with infrastructure dependent on permafrost stability and where ice is needed for travel, hunting and the protection of shoreline from coastal erosion during autumn and winter storms.
- Disruptive impacts on subsistence food species include changes in their range and a possible increase in the transport of some contaminants to the Arctic from source regions at more southerly latitudes, and thus higher levels in top predators. This creates the potential for increased susceptibility of subsistence species to existing and emerging zoonotic pathogens.
- Disruptive climate-mediated impacts on rural drinking water systems and freshwater ponds and lakes include newly arriving species carrying potential water-borne pathogens, harmful algal blooms, and thawing permafrost containment of ponds used for community drinking water.
- Environmental threat assessment methods allow a community to establish existing and emerging threats, and to identify those residents that are most vulnerable to the prioritized threats. With appropriate technical support from the relevant agencies, communities can then develop adaptation strategies for responding to the threats identified. These strategies will focus on reducing exposure and risk. With the various elements of the strategy having been developed, metrics are selected to monitor trends in priority threats, recognise new threats as they emerge and evaluate the success of the adaptation strategy.
- Community-based environmental monitoring networks can help provide the data needed to better understand the effects of global climate change on movement of contaminants and pathogens through the Arctic region.

Gaps in knowledge concerning adaptation strategies for Arctic communities in a warming climate, particularly in terms of food and water security, may be addressed by:

- Continuing the development of culturally specific risk communication.
- Developing regional models of contaminant transport, especially local release from thawing permafrost.
- Improving regional and circumpolar knowledge of the prevalence and trends in zoonotic pathogens in key subsistence species, and identifying intermediate and transport host species for key pathogens.
- Undertaking a systematic international circumpolar evaluation of pathogen and contaminant monitoring protocols, and basic public health adaptation strategies, to ensure the comparability of different monitoring data sets.
